# Scalable Manufacture of Curcumin-Loaded Chitosan Nanocomplex for pH-Responsive Delivery by Coordination-Driven Flash Nanocomplexation

**DOI:** 10.3390/polym14112133

**Published:** 2022-05-24

**Authors:** Ziwei Xia, Zhinan Fu, Li Li, Enguang Ma, Liang Sun, Qinyu Ma, Xuhong Guo

**Affiliations:** 1 State Key Laboratory of Chemical Engineering, East China University of Science and Technology, Shanghai 200237, China; xiaziwei0228@163.com (Z.X.); maqinyu316@163.com (Q.M.); 2 School of Chemistry and Chemical Engineering, Shihezi University, Shihezi 832003, China; maenguang@163.com (E.M.); sunliang055x@163.com (L.S.)

**Keywords:** chitosan, flash nanocomplexation, coordination-driven, curcumin

## Abstract

Metal coordination-driven nanocomplexes are known to be responsive to physiologically relevant stimuli such as pH, redox, temperature or light, making them well-suited for antitumor drug delivery. The ever-growing demand for such nanocomplexes necessitates the design of a scalable approach for their production. In this study, we demonstrate a novel coordination self-assembly strategy, termed flash nanocomplexation (FNC), which is rapid and efficient for the fabrication of drug-loaded nanoparticles (NPs) in a continuous manner. Based on this strategy, biocompatible chitosan (CS) and Cu^2+^ can be regarded anchors to moor the antitumor drug (curcumin, Cur) through coordination, resulting in curcumin-loaded chitosan nanocomplex (Cur-loaded CS nanocomplex) with a narrow size distribution (PDI < 0.124) and high drug loading (up to 41.75%). Owing to the excellent stability of Cur-loaded CS nanocomplex at neutral conditions (>50 days), premature Cur leakage was limited to lower than 1.5%, and pH-responsive drug release behavior was realized in acidic tumor microenvironments. An upscaled manufacture of Cur-loaded CS nanocomplex is demonstrated with continuous FNC, which shows an unprecedented method toward practical applications of nanomedicine for tumor therapy. Furthermore, intracellular uptake study and cytotoxicity experiments toward H1299 cells demonstrates the satisfied anticancer efficacy of the Cur-loaded CS nanocomplex. These results confirm that coordination-driven FNC is an effective method that enables the rapid and scalable fabrication of antitumor drugs.

## 1. Introduction

Cancer, one of the most devastating diseases in the world, is a serious threat to human health today [1]. Chemotherapy is still one of the primary and effective clinical strategies to treat cancer [2]. However, most antitumor drugs are often limited by poor drug water solubility, lack of tissue selectivity and serious toxic side effects [3,4,5]. In recent decades, increasing attention has been paid to nano-based drug delivery systems for tumor therapy. To date, stimuli-responsive drug delivery systems have received considerable research interest in chemotherapy applications due to the improved therapeutic efficacy. Various external stimuli such as pH [6,7,8], redox reagents [9,10], light [11,12] and temperature [13,14] have been widely used to modulate the release and take-up of antitumor drugs to targeted cells. Among them, the pH-responsive delivery system is the most extensively studied, owing to the acidic extracellular pH environment of most cancer tissues [15,16,17].

Currently, the most common routes for pH-responsive delivery systems include constructing pH-responsive bonds and protonation/deprotonation of polymer. It is well-known that the formation and breakage of coordination bonds are sensitive to external pH changes, which can give rise to the potential materials for pH-responsive drug delivery [18]. In this aspect, coordination-driven self-assembly has been gaining attention in biomedical fields [16,19,20].

For example, Li et al. [21] reported an amino acid coordination-driven self-assembly method to prepare curcumin nanodrugs with enhanced biological stability and antitumor activity. Liu et al. [22] developed a coordination self-assembly of natural flavonoids into robust NPs and enhanced stability and solubility of luteolin in vitro cancer therapy. Despite remarkable achievements in coordination-driven self-assembly-based research, there is still a severe shortage of methods that allow the efficient and scalable production of these nanomedicines with high reproducibility.

Recently, flash nanocomplexation (FNC) has been a promising continuous technique to prepare drug-loaded NPs in a scalable and well-controlled manner [23,24,25]. The FNC process accomplishes rapid and efficient mixing of active ingredients in a space-confined microchamber [26]. It allows for the fabrication of a variety of nano-sized particles that undergo self-assembly by metal coordination, electrostatic or hydrogen bond interactions [27,28,29]. Compared with bulk mixing, the FNC process offers greater scalability, higher batch-to-batch reproducibility and better controllability [30,31].

Chitosan (CS), a natural polyelectrolyte, has been widely utilized in delivery carriers due to its low cost, biocompatibility and biodegradability [32,33,34]. Moreover, CS has been well-recognized as a promising biomaterial to coordination-driven self-assembly with metal ions due to the numerous amino and hydroxyl groups [35,36,37].

**Figure 1:**
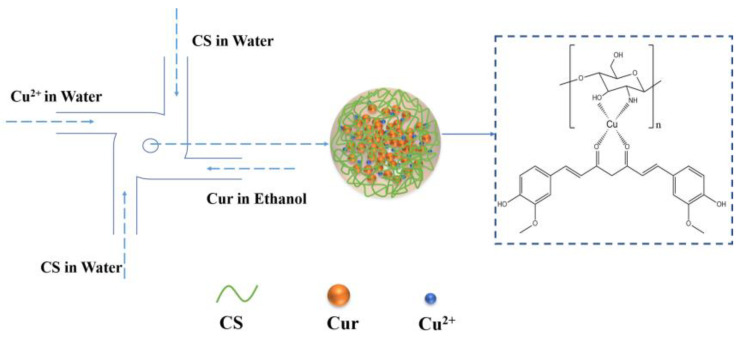
Schematic illustration of FNC turbulent mixing to produce a Cur-loaded CS nanocomplex.

In this work, a simple and scalable FNC technology was utilized to develop a pH-responsive delivery system based on the construction of CS-metal-drug coordination bonding (Figure 1). Cu^2+^ with biocompatibility has been used for coordination, combining CS and drug molecules [38]. Curcumin (Cur) with coordination-bonding-capable diketo/enol moiety has been selected as a model drug [21,39]. Chuah et al. reported that curcumin-containing chitosan nanoparticles (CUR-CS-NP) have improved mucoidhesion compared to unloaded chitosan nanoparticles (CS-NP) [40]. The Cur-loaded CS nanocomplex with controllable size (178–401 nm), high drug loading capacity (41.75%), excellent stability (>50 days) and pH-responsive drug release ability could be easily obtained. Moreover, the scalable production of Cur-loaded nanoparticles was demonstrated with high reproducibility. Furthermore, in vitro evaluations demonstrated that Cur-loaded CS nanocomplex showed enhanced antitumor activity when compared with free Cur toward H1299 cells.

## 2. Materials and Methods

### 2.1. Materials

Chitosan (CS) with an average molecular weight of 6.5 kDa was supplied by Nantong Feiyu Biological Technology Co., Ltd. (Jiangsu, China). Curcumin (Cur, 95.0% purity) was purchased from Adamas-beta (Shanghai, China). Copper chloride (CuCl_2_·2H_2_O), 3 (N-morpholino)-propanesulfonic acid (MOPS) and acetic acid (99.0%) were obtained from Aladdin (Shanghai, China). Sodium hydroxide (NaOH, 99.0%) was obtained from J&K Chemicals. Ethanol and Tween 80 were purchased from Greagent. Ltd. (Shanghai, China). RPMI 1640 medium, fetal bovine serum (FBS), trypsin-EDTA, PBS, 3-(4,5-Dimethyl-thiazol-2-yl)-2,5-diphenyl tetrazolium bromide (MTT), dimethyl sulfoxide (DMSO) and human cancer cell H1299 were purchased from Solarbio science & technology Co., Ltd. (Beijing, China). All chemicals were of analytical grade. Deionized water was obtained from a Milli-Q water purification system and used in all experiments.

### 2.2. Preparation of Cur-Loaded CS Nanocomplex

The Cur-loaded CS nanocomplex was prepared by FNC using a multi-inlet vortex mixer (MIVM). Four inlets were connected to four hermetic stainless syringes via Teflon tubing. CS was dispersed into deionized water with sonication to yield a solution containing 1.0 mg/mL of CS (Stream 1 and 2). CuCl_2_·2H_2_O was dissolved in deionized water at a concentration of 7.0 mM (Stream 3). Cur was dissipated in ethanol with 30 min sonication, and a 0.8 mg/mL solution was obtained (Stream 4). Stream 1 and 2 were fed at a flow rate of 12 mL/min along with the other streams (Stream 3 and 4) fixed at 24 mL/min into MIVM. The free Cur and organic solvent were removed by dialysis. The final product was freeze-dried and kept dry.

### 2.3. Morphology and Structure Characterization

The particle size and size distribution of the nanocomplex were measured by dynamic light scattering performed on a NICOMP 380 ZLS instrument (PSS Inc, Santa Barbara, CA, USA) with a scattering angle of 90°. Transmission electron microscopy (TEM, JEM-1200EX, Tokyo, Japan) was employed to observe the morphology and size of the Cur-loaded CS nanocomplex. Fourier transform–infrared spectrometry (Merck, Darmstadt, Germany) was implemented by a potassium bromide (KBr) tableting method to characterize the intermolecular forces between Cur and metal ion. A UV-vis spectrophotometer (UV-2550) was used to record spectra of Cur-loaded CS nanocomplex from 200–600 nm. Confocal laser scanning microscopy (CLSM, LEICA TCS SP8) was used to capture the fluorescence image of Cur in a tumor cell.

### 2.4. Encapsulation Efficiency and Drug Loading Capacity

The encapsulation efficiency (EE) and drug capacity (DLC) of Cur-loaded CS nanocomplex were determined by the UV-vis spectrophotometer. After removal of the free Cur and residual organic solvent by dialysis, the concentration of Cur in nanocomplex was measured using the UV-vis spectrophotometer at 425 nm. EE and DLC were calculated by the following equations:

$$\mathrm{EE}\;(\%)=\frac{\mathrm{Total}\;\mathrm{amount}\;\mathrm{of}\;\mathrm{loaded}\;\mathrm{CUR}}{\mathrm{Total}\;\mathrm{amount}\;\mathrm{of}\;\mathrm{CUR}\;\mathrm{added}}\;\times \;100$$

$$\mathrm{DLC}\;(\%)=\frac{\mathrm{Total}\;\mathrm{mass}\;\mathrm{of}\;\mathrm{loaded}\;\mathrm{CUR}}{\mathrm{Total}\;\mathrm{mass}\;\mathrm{of}\;\mathrm{NPs}}\;\times \;100$$

### 2.5. Kinetically Control of Cur-Loaded CS Nanocomplex Size

Reynolds number (*Re*) was used to indicate the turbulence degree during mixing [41,42]. The mixing *Re* can be described as [43]:

∑i=1nRei=∑i=1nρiQidsµi

*ρ*_i_ is the fluid density (kg/m^3^), *Q_i_* is the stream flow rate (m^3^/s), *d* is the stream inlet diameter of the mixer (m), *s* is the cross-sectional area of the inlet channel (m^2^) and *μ_i_* is the fluid viscosity (kg/m·s). *d* is 1.1 × 10^−3^ m, and *s* is 1.65 × 10^−3^ m^2^ for all four inlet channels. At room temperature, *ρ_i_* is 1.0 × 10^3^ kg/m^3^ for water and 7.89 × 10^3^ kg/m^3^ for ethanol. *μ_i_* is 8.94 × 10^−4^ Pa· s for water and 1.096 × 10^−3^ Pa s for ethanol.

### 2.6. Stability Test

The stability of the Cur-loaded CS nanocomplex was measured by monitoring the particle size and PDI over 50 days at room temperature using DLS. The Cur-loaded CS nanocomplex was dialyzed against deionized water and then stored in darkness. At regular intervals of time, 1 mL solution was taken out for DLS measurement.

### 2.7. In Vitro Drug Release Profiles

The release behavior of Cur-loaded CS nanocomplex was observed by UV-vis spectrophotometry. In brief, 3 mL of aqueous solution of Cur-loaded CS nanocomplex was transferred into a dialysis bag (7 kDa) and suspended in 30 mL release medium (pH 4.0, 5.0, 6.5 and 7.4) containing 1.0% (*v*/*v*) of Tween 80. The mixtures were placed on a shaker at 37.0 °C and shaken at a speed of 170 rpm. At regular intervals of time, 1 mL of the release medium was collected, and equivalent fresh medium was added to maintain a constant volume.

### 2.8. In Vitro Cellular Uptake

H1299 cells were cultured in RPMI-1640 in a humidified environment at 37 °C and 5% CO_2_. The cell uptake of Cur in different situations was investigated. H1299 cells were seeded in a 35 mm culture dish at a density of 1 × 10^5^ viable cells per well and incubated at 37 °C for 24 h to allow the cells to attach. The cells were incubated for 1, 2, 4 and 6 h in fresh medium containing Cur-loaded CS nanocomplex (Cur concentration was 20 μg/mL). Fluorescence images were collected using a laser confocal microscope (LEICA TCS SP8).

### 2.9. In Vitro Antitumor Activity

Cell viability was quantified by the MTT method. H1299 cells were seeded in 96-well plates (1 × 10^4^ cells/well) and incubated at 37 °C for 24 h in an incubator. The medium was then replaced with fresh medium containing Cur-loaded CS nanocomplex and pure Cur to make a final Cur concentration of 1, 10 and 20 ug/mL. Subsequently, H1299 cells received further incubation for 24 h. Cell viability of CS-metal and pure Cur against H1299 cells at 24 h was evaluated as a control. The cell viability was calculated according to the following equation:

$$\mathrm{Cell}\;\mathrm{viability}\;(\%)=\frac{\mathrm{Absorbance}\;\mathrm{of}\;\mathrm{test}\;\mathrm{cells}}{\mathrm{Absorbance}\;\mathrm{of}\;\mathrm{control}}\;\times \;100$$

The data were expressed as mean ± standard deviation. Duncan test was used for statistical comparison. *p* < 0.05 was considered to be significant.

## 3. Results

### 3.1. Preparation and Characteristic of Cur-Loaded CS Nanocomplex

**Figure 2:**
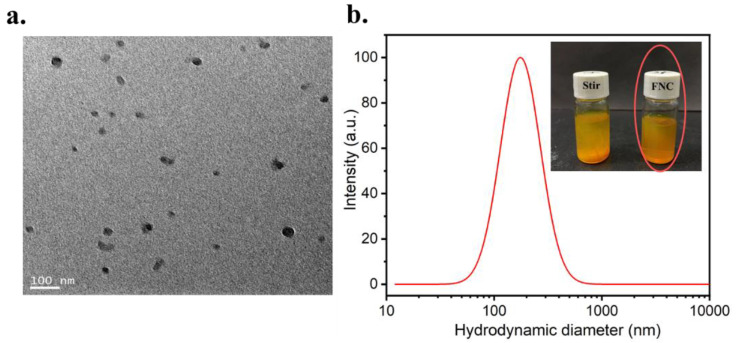
(**a**) TEM image of the Cur-loaded CS nanocomplex. (**b**) DLS profiles with pictures of the samples shown in the inset.

Cur-loaded CS nanocomplex was prepared by a rapid mixing of Cur, Cu^2+^ and CS using an FNC apparatus. As shown in Figure 2a, the Cur-loaded CS nanocomplex showed a spherical structure without obvious aggregates. In Figure 2b, the nanocomplex prepared by FNC showed a suitable particle size (190 ± 5 nm) and narrow size distribution (PDI = 0.124 ± 0.049), which benefits the enhanced permeability and retention (EPR) effect [44]. As a comparison, another experiment with conventional mixing was carried out. First, 3 mL of CS and 3 mL of Cu^2+^ were added into a flask and stirred with a magnetic stirrer for 1 h. Then, 3 mL of Cur was added into the flask and stirred for 4 h. However, an unstable suspension with particles visible to the naked eye was obtained via a conventional mixing method (Figure 2b), and the particle size was in the range of a micrometer (ca. 1800 nm, PDI = 0.789).

**Figure 3:**
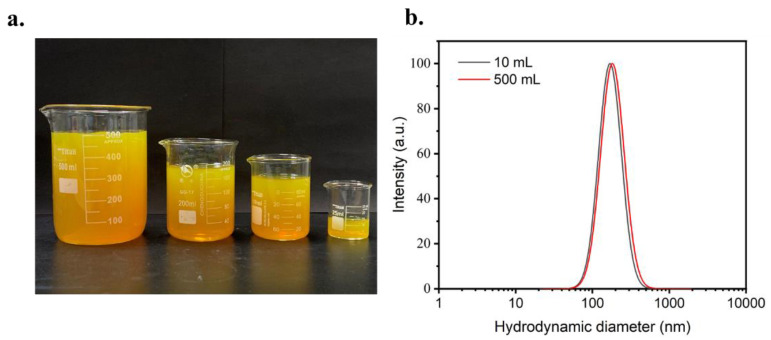
(**a**) Digital photograph of Cur-loaded CS nanocomplex solutions with different volumes of (10, 100, 200 and 500 mL) prepared FNC. (**b**) DLS results of samples with cumulative volumes of 10 and 500 mL by continuous FNC operation.

In addition, a remarkable advantage of FNC over the conventional method is the continuous operation mode, which enables the scalable production of the Cur-loaded CS nanocomplex. The production volume of the Cur-loaded CS nanocomplex was multiplied from 10 to 500 mL (Figure 3a). As shown in Figure 3b, the DLS data clearly showed the high reproducibility for nanocomplex formation in FNC. These results indicated that the FNC technique was a promising method to produce nanomedicine on scale.

**Figure 4:**
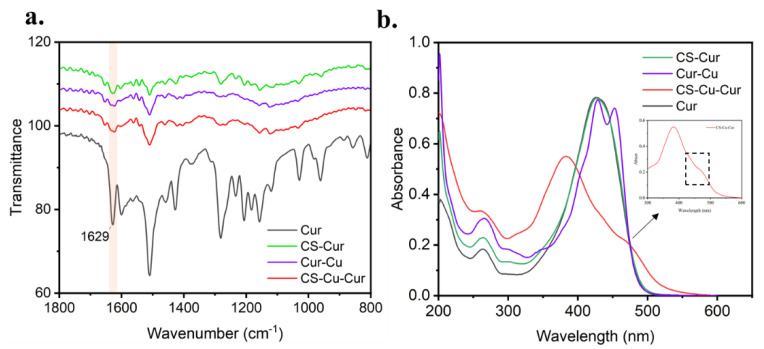
(**a**) Fourier transform infrared spectroscopy (FT-IR) and (**b**) UV-vis absorption spectra of CS-Cur, Cur-Cu, Cur-loaded CS nanocomplex, Cur and CS-Cu.

The formation of the coordination bond between CS and Cu^2+^ as well as Cur and Cu^2+^ may be confirmed by FT-IR and UV-vis spectra. As shown in Figure 4a, the typical band at 1629 cm^−1^ indicated a stretching band of the carbonyl group in Cur [4]. Seen from the FT-IR spectrum of Cur-Cu, CS-Cur and CS-Cu-Cur, the shift to lower frequency of carbonyl group stretching vibration reveals that the coordination interaction may take place between Cu^2+^ (or CS) and Cur [45].

In addition, the formation of the coordination bond between ligands and Cu^2+^ is also confirmed by UV-vis spectra (Figure 4b). Cur-loaded CS nanocomplex showed a shoulder peak at 453 nm, which may originate from the charge transfer between Cur and Cu^2+^ (Cur-Cu shows an obvious shoulder at 453 nm) [46], whereas neither pure Cur nor CS-Cur were observed in UV-vis spectra [21]. These results indicated that coordination interaction of ligands to Cu^2+^ occurs in the formation of the Cur-loaded CS nanocomplex.

### 3.2. Encapsulation Efficiency (EE) and Drug Loading Capacity (DLC)

**Figure 5:**
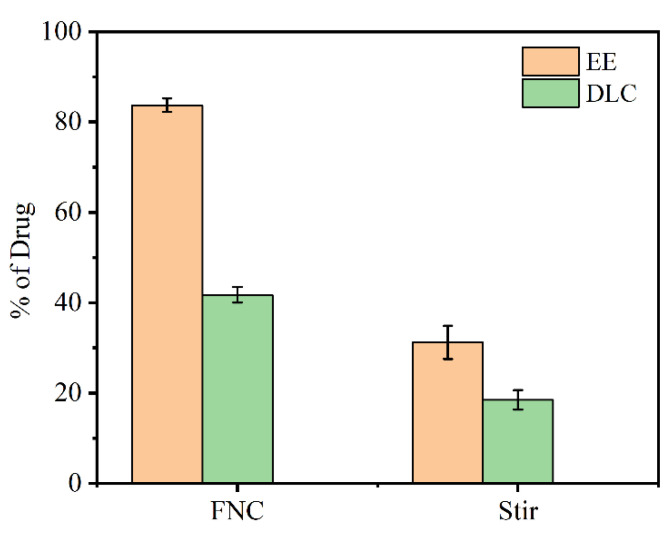
Encapsulation efficiency and drug loading capacity of Cur-loaded CS nanocomplex prepared by FNC and conventional bulk methods.

The encapsulation efficiency and drug loading capacity of Cur-loaded CS nanocomplex obtained via different methods were calculated, respectively. As seen in Figure 5, the FNC-produced sample afforded an encapsulation efficiency of 83.71% ± 1.50% and drug loading capacity of 41.75% ± 1.75%, which were much higher than those of samples prepared by the conventional bulk method (EE = 31.19 ± 3.67 and DLC = 18.48 ± 2.12). It suggested that FNC-prepared Cur-loaded CS nanocomplex will be an efficient drug delivery system.

### 3.3. Stability of Cur-Loaded CS Nanocomplex

**Figure 6:**
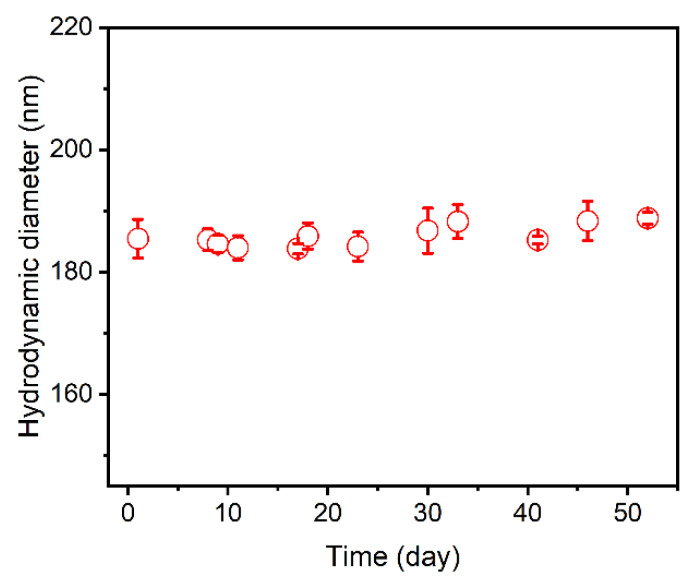
The time dependent size stability of Cur-loaded CS nanocomplex.

For a clinical application of the antitumor drug, its stability in aqueous solution is of great importance. Figure 6 shows the size stability of the Cur-loaded CS nanocomplex generated by the FNC method. The Cur-loaded CS nanocomplex kept stable in terms of the particle size and size distribution for 50 days, which confirmed that the Cur-loaded CS nanocomplex is highly stable in an aqueous medium.

### 3.4. Size Control of Cur-Loaded Particles by Reynolds Number

**Figure 7:**
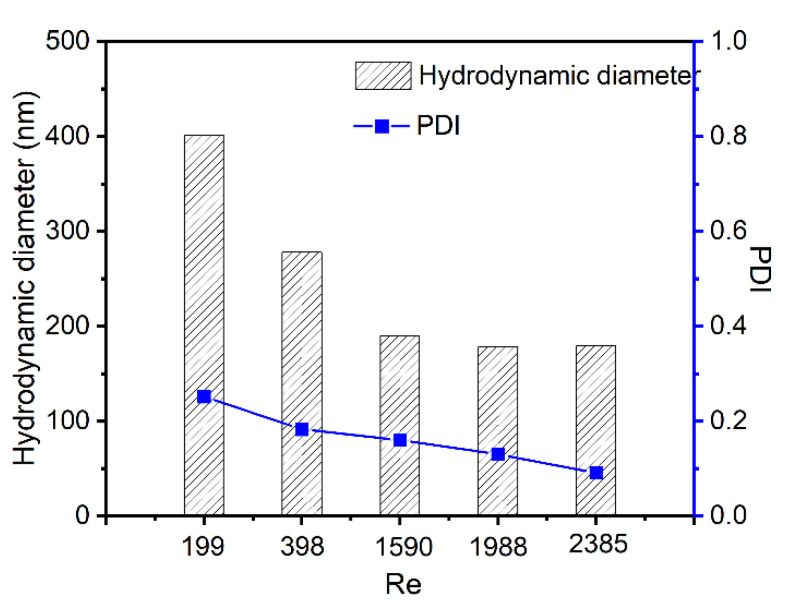
Effect of *Re* on the average particle size and polydispersity index (PDI) of Cur-loaded CS nanocomplex.

One key advantage of the FNP technique is the controllable process, which can readily tune the size of Cur-loaded CS nanocomplex by changing the *Re* number [47,48,49]. A set of *Re* numbers were tested when other parameters remained constant. As shown in Figure 7, the sizes of Cur-loaded CS nanocomplex decreased from 400 nm to 190 nm when the *Re* ranged from 199 to 2385. It was observed that the particle size did not further decrease when the *Re* exceeded 1590, which was owing to the homogenous mixing in the FNC process when *Re* reaches a certain degree [50,51].

### 3.5. Profiles of Curcumin Release at Different pH

**Figure 8:**
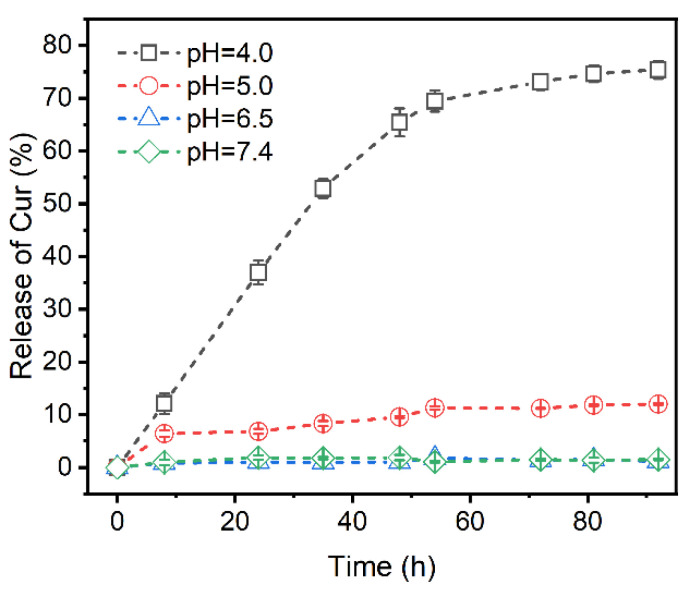
Release profiles of Cur-loaded CS nanocomplex at different pH.

As is known, the formation and cleavage of the coordination bond are sensitive to external pH variations [18,52]. The in vitro release of Cur from the Cur-loaded CS nanocomplex in release mediums of different pH values has been investigated (Figure 8). Under the physiological condition (pH 7.4), the Cur-loaded CS nanocomplex showed a slow-release profile and about 1.5% of the Cur was released from the nanocomplex within 50 days. However, significant release of Cur from Cur-loaded CS nanocomplex could be observed when the pH of the environmental medium was decreased (pH 4.0), confirming the high sensitivity of Cur-loaded CS nanocomplex to pH stimulus. This pH-responsive ability suggests that Cur-loaded CS nanocomplex can be regarded as a controllable drug delivery system for the treatment of tumor therapy.

### 3.6. Cellular Uptake Study

**Figure 9:**
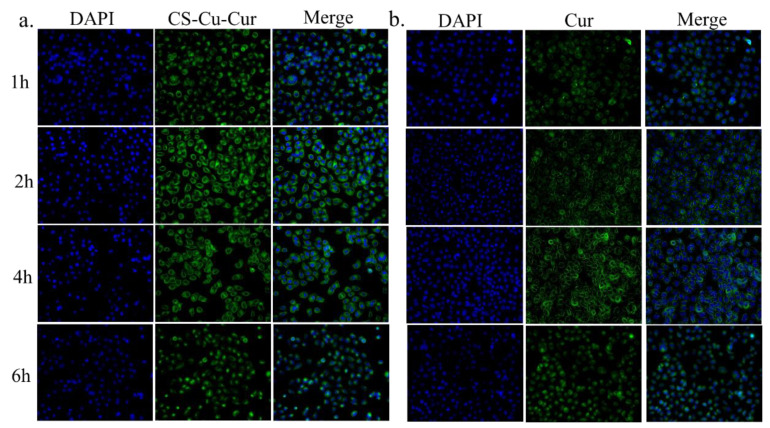
Confocal laser scanning microscope images of intracellular uptake of (**a**) Cur-loaded CS nanocomplex and (**b**) pure Cur by H1299 cells. Concentration of Cur is 20 ug/mL.

It has been intensively investigated that effective cellular internalization is a requirement for antitumor drug carriers [28,53]. To observe the penetration of Cur-loaded CS nanocomplex and pure Cur, the distribution of Cur in H1299 cells was monitored after incubation for 1, 2, 4 and 6 h under a confocal laser scanning microscope (CLSM). As shown in Figure 9, the green fluorescence appeared in the cytoplasm for cells treated with pure Cur or Cur-loaded CS nanocomplex after incubation for 1 h. After being incubated with Cur-loaded CS nanocomplex and pure Cur, the fluorescence signal of Cur-loaded CS nanocomplex was always stronger than that of pure Cur, which suggested that Cur-loaded CS nanocomplex has a more effective delivery of Cur into the cells compared to pure Cur. For the Cur-loaded CS nanocomplex, cellular uptake reached a peak in 2 h, but pure Cur reached the peak in 4 h.

### 3.7. In Vitro Cytotoxicity Study

**Figure 10:**
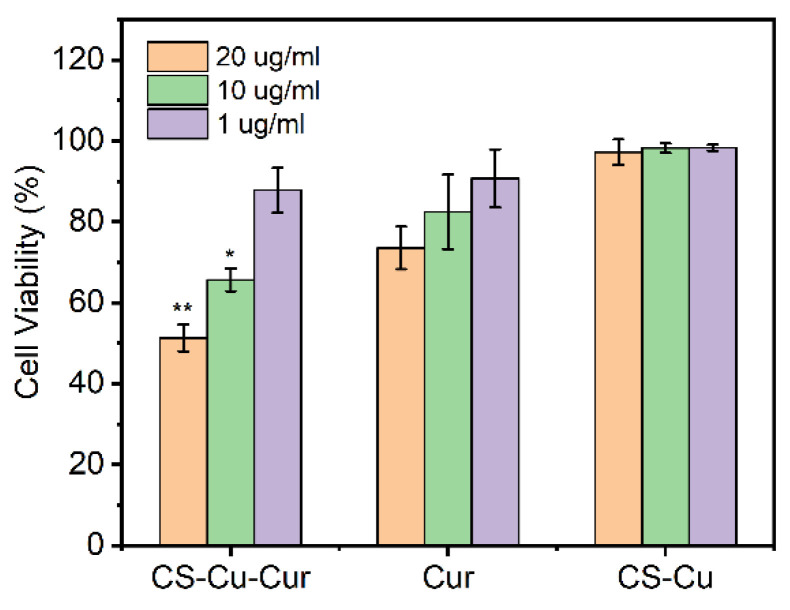
In vitro cytotoxicity of the Cur-loaded CS nanocomplex, free Cur, and CS-metal complex against H1299 cells incubated for 24 h. In all panels, the indicated concentrations are Cur doses. It should be noted that for evaluating Cur-loaded CS nanocomplex, equal concentrations of CS-metal complex were employed to eliminate the effect of vehicles in MTT assay. Error bars denote the standard deviation (n = 6). * and ** indicate significant *(p* < 0.05) and highly significant (*p* < 0.01) differences between Cur-loaded CS nanocomplex and free Cur.

The cytotoxicities of Cur-loaded CS nanocomplex, CS-Cu and pure Cur against H1299 cells for 24 h were evaluated by MTT assay. As shown in Figure 10, it showed that CS-Cu as a drug carrier exhibited no significant cytotoxicity. After H1299 cells were co-incubated with Cur-loaded CS nanocomplex and pure Cur, they displayed a dose dependence on the cytotoxic effect when three concentrations were tested. Compared with pure Cur, Cur-loaded CS nanocomplex apparently exhibited a better cell proliferation inhibition effect with *p* < 0.05 and *p* < 0.01 at a concentration of 10 and 20 µg/mL, respectively (Figure 10). As these results showed, Cur-loaded CS nanocomplex considerably enhanced the biological effect of Cur, suggesting that Cur-loaded CS nanocomplexes have great application potential in antitumor therapy.

## 4. Conclusions

In summary, pH-responsive Cur-loaded CS nanocomplex were successfully prepared based on construction of CS-Cu^2+^-Cur coordination-bonding using the FNC method. The approach is a convenient, effective and scalable process with high reproductivity. The size of Cur-loaded CS nanocomplex is tunable by controlling the mixing of Re. The optimized Cur-loaded CS nanocomplex performed a narrow size distribution (PDI = 0.124 ± 0.049), high drug-loading capacity (41.8 ± 1.8%) and pH-responsive drug release behaviors. The results observed by CLSM demonstrated Cur-loaded CS nanocomplex has more effective and faster cellular uptake of Cur molecules into cancer cells compared to pure Cur. Furthermore, Cur-loaded CS nanocomplex showed a higher cytotoxicity against H1299 cells than pure Cur. These findings demonstrated that the coordination driven FNC technique opens a new way for scalable manufacture of antitumor nanoparticles.

## Data Availability

The data presented in this study are available on request from the corresponding author.
